# Vaccine cold chain and understanding what underpins vaccine security for vaccine preventable diseases

**DOI:** 10.1136/bmjmed-2025-001835

**Published:** 2026-04-09

**Authors:** Gilbert Rukundo, Matthew Moore, Herve Semukunzi, Sudeshna Chatterjee, Jean Pierre Musabyimana, Claude Mambo Muvunyi, Christopher Aird Green

**Affiliations:** 1 School of Chemical Engineering, College of Engineering and Physical Sciences, University of Birmingham, Birmingham, UK; 2 University of Rwanda, Kigali, Rwanda; 3 Rwanda Biomedical Centre, Kigali, Rwanda; 4 Africa Centre of Excellence for Sustainable Cooling and Cold Chain (ACES), Kigali, Rwanda; 5 University Hospitals Birmingham NHS Foundation Trust, Birmingham, UK

**Keywords:** Public health, Infectious disease medicine, Community health services

## Abstract

Vaccines have saved an estimated 154 million lives in the past 50 years and support 15 of the 17 United Nations sustainable development goals. Vaccines are also an important tool in the control of new outbreaks of infectious diseases. The vaccine cold chain, however, is key in enabling and empowering implementation of vaccine policy and societal protection from all vaccine preventable diseases, and is especially relevant in low and middle income countries in sub-Saharan Africa. The vaccine cold chain is a complex, highly specialised, temperature controlled supply chain network that extends from the point of vaccine manufacture to dose administration, and has multiple points of vulnerability. Large quantities of vaccines are lost because of excess heat or accidental freezing, resulting in missed opportunities for vaccination. Disruption to the provision of routine vaccines during the covid-19 pandemic resulted in millions of children not being vaccinated. The vaccine cold chain needs strategic prioritisation for investment and innovation so that the next generation of vaccine cold chains for low and middle income countries can be designed towards providing reliable and sustainable vaccine security in an uncertain world of climate change, managing the advent of new vaccine technologies, and narrowing inequalities in global health for resource poor communities. This review focuses on the vaccine cold chain in African low and middle income countries, and how new and emerging advances in vaccine science and challenges will affect the readiness to control the burden of vaccine preventable disease on the continent.

## Introduction

 In May 1974, the World Health Organization (WHO) introduced the Expanded Programme of Immunisation to reduce morbidity and death from vaccine preventable disease. Originally targeting six pathogens (tuberculosis, poliomyelitis, diphtheria, tetanus, pertussis, and measles), by 2010 the list had expanded to include hepatitis B virus, mumps virus, rubella virus, *Haemophilus influenzae* type B, varicella virus, rotavirus, pneumococcus, and human papillomavirus, with more additions to follow in later years. Global coverage of WHO Expanded Programme of Immunisation, as measured by the third dose of the diphtheria-tetanus-pertussis vaccine, increased from <5% in 1974 to 86% in 2019 before the covid-19 pandemic.^1^ By 2024, marking 50 years from the inception of the WHO Expanded Programme of Immunisation, vaccines for 14 target pathogens had saved about 154 million lives globally.^2^ This finding translates to one life saved every 10 seconds, of whom 146 million and 101 million were children aged <5 years and infants, respectively. For each death prevented, an average of 66 years of full health were gained and vaccination accounted for 40% of the observed reduction in global infant mortality (52% in WHO Africa region).^2 3^ Unicef alone procures >2 billion WHO Expanded Programme of Immunisation vaccine doses globally each year as a central pillar of public health, and global vaccine production is about seven billion doses (2024 WHO Global Vaccine Market report). Vaccinations affect 15 of the 17 United Nations sustainable development goals and are central to societal prosperity.

No lives would be saved by a vaccine, however, without robust and reliable cold chains to underpin implementation of vaccine policy. The vaccine cold chain is central to a wider healthcare ecosystem that encompasses financing, procurement, specialist workforce skills, data, and community engagement. The vaccine cold chain is independent of vaccine product and disease target, and hence underpins the empowerment and effect of all vaccination policies in all regions of the world. The vaccine cold chain is a critical public health national infrastructure that protects society from all vaccine preventable diseases and is comparable with energy, defence, transportation, and other systems needed for a functional and prosperous future. In this review, we focus on vaccine cold chains in low and middle income countries in sub-Saharan Africa, with an emphasis on the future and next generation of the vaccine cold chain that will be needed for sustainable and reliable access to vaccines and to reduce global inequalities in health.

## Vaccine cold chain

**Figure 1:**
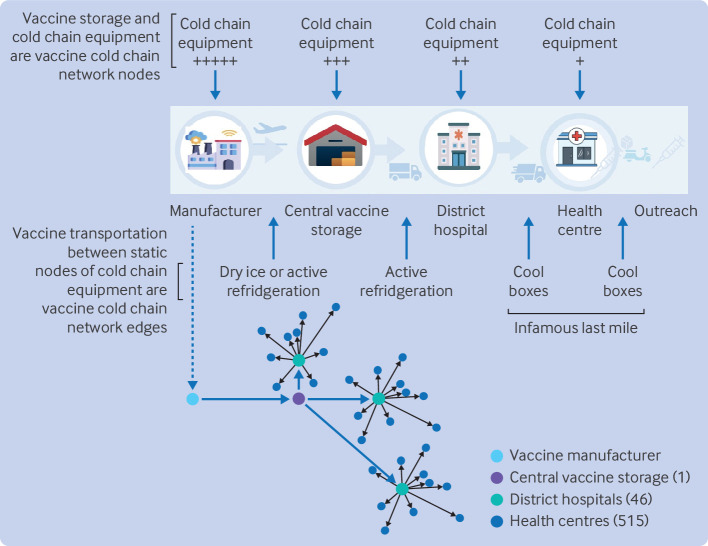
Depiction of vaccine cold chain in African low and middle income countries. The vaccine cold chain is a continuous, uninterrupted, monitored, temperature controlled supply chain from vaccine manufacture to dose administration (top panel). Vaccine fridges and freezers at health facilities meeting the World Health Organization (WHO) standards for performance, quality, and safety, act as storage nodes in the network (or cliques when several fridges or freezers are co-located). Vaccines are distributed between nodes by active and passive cooling transportation with WHO performance, quality, and safety approved monitoring and standard operating procedures. The chain is replicated across every path of the vaccine cold chain network. With Rwanda as an example (bottom panel), vaccines are imported directly from the manufacturer by aircraft and enter the first vaccine cold chain node (or clique) in the vaccine cold chain network for low and middle income countries. From here, a complex combination of push and pull models supply vaccines when required through the first set of network edges within the country to district hospital level nodes (or cliques). The final tier is supply to local health centres and the infamous last mile challenges where vials will be opened in vaccination clinics. Numbers denote the quantity of each type of node in Rwanda at the time of writing. Outreach activities to small remote communities are relatively infrequent and most vaccination doses are given at regular vaccination clinics at the health centre level

Every vaccine cold chain is essentially a complex network and spatial distribution of dedicated cold chain equipment interconnected by temperature controlled transportation logistics, under continuous management oversight for major deviations in temperature control, vaccine expiry, and stock distribution (figure 1). The main function of the vaccine cold chain is to preserve the biological potency of vaccine products that have been licensed as safe and effective from clinical trials, and to simultaneously distribute these in a timely way to the many wide and distant points of need. Even temporary changes in temperature during the storage or transportation of vaccines can irreparably damage the product, rendering the vaccine ineffective in providing immunity when needed.

Based on network analysis methodology and terminology, each piece of static vaccine cold chain equipment (eg, a fridge) can be considered a node and the dots on a network map, with each node having different properties or attributes, such as storage capacity, energy security, vaccine stock, and maintenance needs. Transportation of vaccine stocks between nodes are considered edges and are the lines between nodes on network maps that describe how nodes are connected to each other. Each edge will have certain properties or attributes, such as distance or time, and whether active or passive cooling and temperature monitoring are used. The whole vaccine cold chain for a country can have many hundreds or thousands of interconnected nodes and edges; some will be functionally or strategically more important than others, and many unique supply chains to individual clinics and regions will have dependencies that can become rate limiting in different contexts that affect the efficient implementation of the national vaccination policy.

Rwanda, to give perspective, is a sub-Saharan African nation of 13.2 million people and is a landlocked country of 26 338 km^2^ in East Africa. More affectionately known as the land of a thousand hills, Rwanda has complex logistical challenges in the vaccine supply chain. The Rwanda vaccine cold chain is managed by one national health implementation agency (Rwanda Biomedical Centre) and is typical of many African countries in featuring a multilevel network of vaccine distributions that has achieved, and sustained, high levels of routine vaccine coverage (>97%, data from 2018)^4^. Data from the 2022 national audit of the vaccine cold chain supply showed that to achieve this level of coverage, 1003 vaccine fridges meeting WHO standards for performance, quality, and safety, were networked across 511 separate supply chains to health centres hosting vaccination clinics, with >4.4 million vaccine doses passing through these networks to protect Rwandan citizens each year.^4 5^

**Figure 2:**
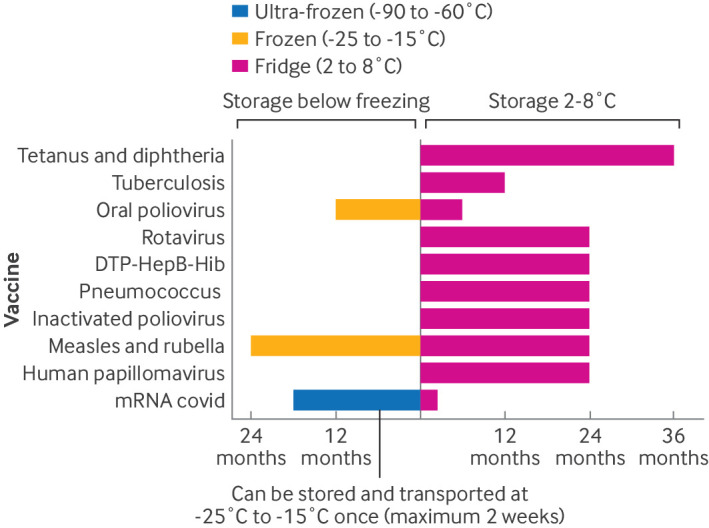
Unopened vaccine stability and storage limitations in World Health Organization (WHO) performance, quality, and safety approved freezers and fridges. This illustration includes vaccine products used in the WHO Expanded Programme of Immunisation schedule in Rwanda at the time of writing, and the manufacturer summary of product characteristics to show the variation in vaccine storage needs and manufacturer specified expiry limitations for unopened vaccine vials. Vaccines were WHO Expanded Programme of Immunisation vaccines for tetanus and diphtheria, tuberculosis (BCG), polio (oral poliovirus and inactivated poliovirus), combined diphtheria, tetanus, pertussis, hepatitis B virus, and *Haemophilus influenzae* type B (DTP-HepB-Hib), pneumococcus (pneumococcal conjugate 13), combined measles and rubella, rotavirus, human papillomavirus, and SARS-CoV-2 virus (covid). The lipid nanoparticle mRNA covid-19 vaccine is shown for perspective of the needs of new genetic vaccines, although not currently being used in Rwanda, with data from the summary of product characteristics for the current Comirnaty vaccine (Pfizer, omicron XBB 1.5)

Biological products such as vaccines share a common vulnerability in needing continuous, uninterrupted, monitored temperature control from the point of manufacture to dose administration,^6 7^ although not all products are the same (figure 2). The target range for storage and distribution for all WHO Expanded Programme of Immunisation vaccines is typically between +2°C and +8°C, although some vaccines can be frozen. Vaccines are transported under active or passive cooling and are stored within this dedicated vaccine distribution network that use cold chain equipment, such as fridges or freezers, vaccine carriers, and temperature monitoring that meet the WHO standards for performance, quality, and safety.

The WHO performance, quality, and safety approval process includes a rigorous technical evaluation and cycle of ongoing, real time reporting of equipment failures, complaints, or changes to maintain performance, quality, and safety.^8^ A WHO approved vaccine fridge typically costs US$3000-5000 (£2228-3715; €2580-4301), which is 3-10 times more expensive than a typical household appliance of comparable storage volume, when designed for accurate temperature stability, protection from freezing, remote temperature monitoring, and resilience to total power failures and extreme external conditions. Superior equipment performance is key.^9^ A typical WHO approved vaccine fridge can maintain a target temperature of 2-8 °C for 72 hours, even in the event of a total power failure if the door remains closed, but during this time vaccination activity must stop until the engineers can fix the problem. Vaccines for human use should also be stored separately from other products to minimise opening of the fridge or freezer door and human errors in dose administration. Case reports of administering insulin in error for the influenza vaccine^10 11^(in the US in 2014 and 2019) and fatal outcomes from muscle relaxant used as a diluent for vaccine preparation (in Syria in 2014 and in Samoa in 2018), as well as other similar safety incidents, highlight the need for strict protocols to regulate the separation, labelling, and handling of vaccines for human use.^12 13^ The vaccine cold chain is not a generic health supply chain but one that is purposely designed and highly specialised.^14^

## Empowerment of vaccine policy

**Figure 3:**
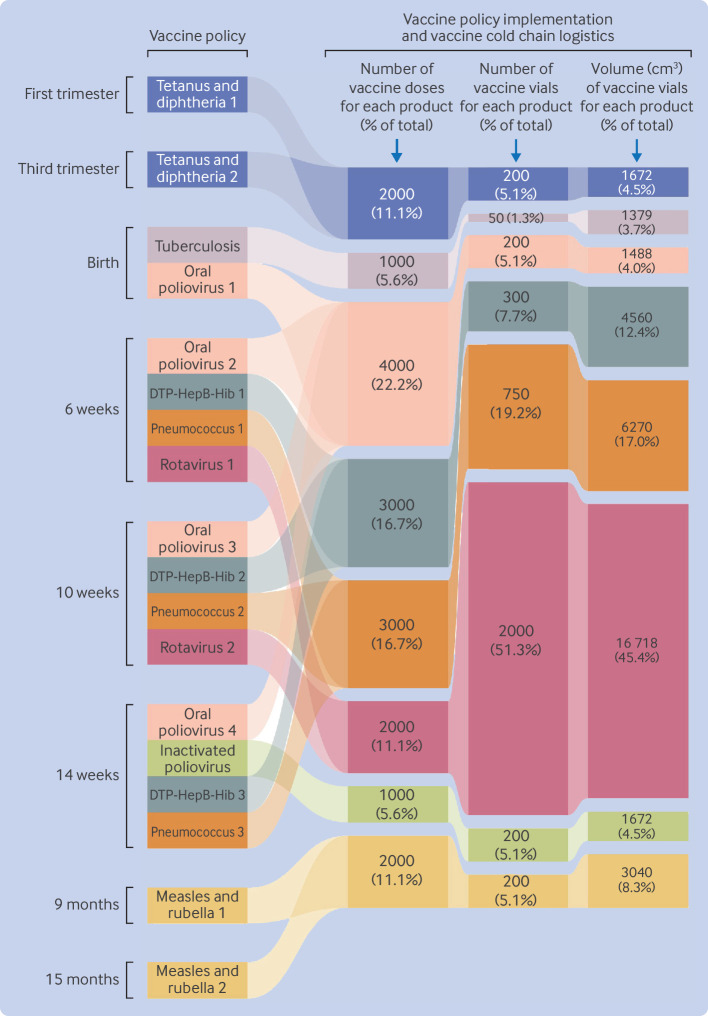
Schematic illustration of translation of vaccine policy to vaccine policy implementation logistics for the World Health Organization (WHO) Expanded Programme of Immunisation schedule, as exemplified by current practice in Rwanda for 1000 infants between pregnancy to 15 months of life and without adjustment for vaccine losses. Vaccines were WHO Expanded Programme of Immunisation vaccines for tetanus and diphtheria, tuberculosis (BCG), polio (oral poliovirus and inactivated poliovirus), combined diphtheria, tetanus, pertussis, hepatitis B virus, *Haemophilus influenzae* type B (DTP-HepB-Hib), pneumococcus (pneumococcal conjugate), and combined measles and rubella. The first column shows the prescribed order of vaccine doses, re-expressed as dose frequencies of each vaccine dose (second column) in the order in which they first appear in the schedule of immunisation. Vaccine manufacturing of different numbers of doses in each vial, however, creates a new logistical picture in determining the number of vials (third column) and volumes used in the vaccine cold chain (final column) for each vaccine product

Morbidity and mortality from vaccine preventable diseases are most severe in the first weeks and months of life. The use of polyvalent (multiple vaccine antigens within one injection) and concurrent (antigens given separately but at the same time) vaccination dosing allows WHO Expanded Programme of Immunisation schedules (with the example of Rwanda) to link protection to 11 separate vaccine preventable diseases from only eight visits to vaccination centres in the first 15 months of life, including two visits in pregnancy. Vaccine manufacturing, however, can substantially change the vaccine logistics demand from the initial recommended order in the schedule of immunisation in the vaccine policy. Vaccine products are sometimes manufactured as single dose vial formulations (eg, rotavirus vaccine) whereas others are multidose (eg, 20 doses in each vial for the BCG vaccine). The combination of vaccine science that recommends the number and intervals for each vaccine dose in the WHO Expanded Programme of Immunisation schedule (and ultimately vaccine policy) and the number of doses manufactured in each vial and vial volumes creates a very different logistical mission for the vaccine cold chain. As shown in figure 3, translation of vaccination policy to policy implementation means that >70% of the total volume of vaccine vial storage in the vaccine cold chain is dedicated to just two vaccine preventable diseases in the first 15 months of life (rotavirus and pneumococcus).

## Vaccine damage, loss, and the infamous last mile

With the focus on African communities, the situation is especially confounded by fragile last mile logistics because of ageing cold chain equipment, insecure grid electricity or diesel generators, or both, and a lack of technicians to service this equipment.^415^,^17^ An estimated ≥25% of vaccine doses are compromised by failures in cold chain custody (freezing or exposure to excess heat) or incomplete vial usage. As a result, 20% of African children do not receive a complete immunisation schedule, and >30 million children aged <5 years have vaccine preventable diseases every year (more than half a million deaths in Africa, corresponding to 58% of global vaccine preventable deaths).^18^ The global cost of vaccine wastage caused by products being exposed to temperatures outside their recommended range is estimated at $34.1bn annually, but other estimates suggest that every dollar spent on child immunisation provides $44 worth of economic benefits in low and middle income countries, with different complex measures of true cost effectiveness.^19 20^

For most vaccines, long term stability under vaccine specific fridge or freezer controls gives 12-36 months of stability for correctly stored unopened vials, providing clinics with the time needed for vaccine distribution and resilience in stockpiling. With only one major temperature deviation, many thousands of doses could be lost, sometimes without healthcare staff being aware or being sufficiently trained to manage this crisis.^21^ Furthermore, in the absence of any recorded temperature deviation, definitively determining in the clinic whether a vaccine product has been compromised is not always possible.

Excess heat can irreversibly change the protein antigen configuration or shape (denaturation) that is essential for correct epitope presentation and antibody recognition, cause breakage of polysaccharide bonds to protein carrier molecules (adjuvant) that is needed for T cell dependent antibody responses and durable immunity, and reduce the viability of already attenuated virus used in live attenuated vaccine preparations. In 1996, as a test for excess heat exposure, a small, thermochromatic label called a vaccine vial monitor was attached to each vaccine vial cap. Vaccine vial monitors use a light coloured and temperature sensitive square superimposed on a larger, dark, circular background for reference. With excess heat exposure, the colour of the square irreversibly darkens to match the background to the naked eye, allowing healthcare staff to identify and discard heat compromised vials. An estimated 200-250 million vaccine vial monitors are used globally each year^22^ with different monitors calibrated for different heat sensitivities; at 37 °C, vaccine vial monitor 2 (most sensitive) will reach colour undifferentiation by 48 hours for most heat sensitive products, such as live oral poliovirus vaccine, and vaccine vial monitors 7, 14, and 30, as the labels suggest, reach the same endpoint at seven, 14, and 30 days, as needed for other products. Vaccine vial monitors cannot signal excess cold or freezing, however, and their response to cumulative heat is delayed, which means recent and brief episodes of excess heat may go undetected. The monitors also add cost and require training of healthcare staff.

Excess cold or freezing can be as dangerous as heat for many products and is alarmingly common, affecting up to 30-40% of stock, and can happen at several points in the vaccine cold chain.^14 16^ At temperatures below zero, protein or polysaccharide subunit conjugates can undergo irreversible changes with adjuvant aggregation and precipitation, structural changes to the protein antigen itself, and cause sedimentation or layering of the vaccine (sometimes visible by the shake test because of the precipitation time of previously frozen vaccines being more than four times faster) that not only undermine immunogenicity but can also increase reactogenicity.^23^

Finally, in the closing minutes of a clinic and at the end of the infamous last mile, when a vial has been opened, the remaining unused doses cannot always be returned to the fridge or freezer in compliance with the original manufacturer’s expiry date. A new stability window applies to opened vaccine vials, and although this window can vary with different products, some vaccines, such as the live attenuated BCG and combined measles-rubella vaccines, are especially vulnerable to incomplete vial usage and open vial losses if not enough children have attended the clinic to use all of the doses in a vial on the same day.^24 25^ BCG is manufactured as 20 doses in each vial, meaning that unused doses are common at the end of a vaccination clinic which, along with the requirement to use or discard a BCG vial within 4-6 hours of opening, regardless of whether the vaccine has been returned to the fridge, causes high wastage. In the context of BCG, local operating procedures to not open a BCG vial for <10 newborn babies are not uncommon, despite this time being the only time point to vaccinate newborn babies.^26^,^28^

## Readiness for public health emergencies

Existing vaccine cold chains in low and middle income countries are mainly designed to deliver the WHO Expanded Programme of Immunisation schedule for routine use in pregnant mothers, infants, and children, scaled by the birth rate with adjustments for vaccine wastage. Vaccine cold chains are less engineered for sudden, unexpected additional demand in the form of supplementary immunisation activity (also sometimes called catch-up or emergency vaccination campaigns), or have redundancies built in for a sudden loss of capacity, such as equipment or energy failures. The disruption caused by the covid-19 pandemic and immunity gaps from missed childhood vaccinations has already resulted in widespread outbreaks of measles, with outbreaks of other avoidable vaccine preventable diseases, such as polio, expected to follow. Even before the covid-19 pandemic, shocks to fragile vaccine cold chain infrastructure resulting in infants and children missing their opportunity for lifelong protection to many vaccine preventable diseases was evident. With measles as an example, during the Ebola virus outbreak in West Africa, the effects on social displacement and priority given to deploying a vaccine for Ebola virus disease coincided with about 7000 deaths from measles compared with 2243 deaths from Ebola virus.^29^ Current vaccine cold chain design, in practice, operates as a staged decompression arrangement to contain the large amount of vaccine stock delivered in one flight (often containing hundreds of thousands of vaccine doses) through to the daily steady rate of a few dozen doses of vaccine given each day at vaccination clinics, but the need to retain the ability to expand the provision of vaccines at short notice, where and when needed to respond to special supplementary immunisation activity. Sustainable resilience is key, and as well as the vaccine cold chain infrastructure, a trained workforce and institutional knowledge on how to best use and maintain this strategy is needed.

Targeted vaccination initiatives represent vaccine cold chain demands over and above the steady state provision of maternal and child health protection. Precise quantities of vaccine used for supplementary immunisation activities are difficult to verify but are estimated to require several million doses of additional vaccine each year to quickly plug immunity gaps in the population without advance knowledge of the target or scale, or where and when this supplementary immunisation activity will be. For the covid-19 pandemic, after the first vaccines were approved in late December 2020, about 5.5 billion people worldwide had received one or more vaccine doses against the SARS-CoV-2 virus by the end of 2021 and in the first 12 months of deployment of the vaccine.^30^ Other notable unscheduled vaccine cold chain demands include the control of outbreaks of Ebola (West Africa, 2014-16),^31 32^ mpox (East Africa, ongoing), and Marburg (Rwanda, 2024) viruses.^33^

A lesser recognised, more widespread, and major demand for supplementary immunisation activity, however, comes not from new pathogens but from missed doses and inadequate immunity resulting from vaccine damage or host factors, such as malnutrition in childhood. Measles virus is a leading example, with a basic reproductive number or R_0_ of 12-18, making it a highly infectious virus that requires a 95% population immunity target threshold for control. Widespread outbreaks of measles have been common across the African continent, especially in the years after the covid-19 pandemic, including in countries previously declared to be measles free, with almost all deaths in children who had not received any routine vaccinations. Similar outbreaks of measles were seen in high income countries, with 37 countries (an increase from 22 countries in 2021) reporting large and disruptive outbreaks.^21 34^ In 2022, an estimated 136 000 people died from measles, an increase of 43% from 2021, because of challenges in vaccine uptake and coverage, according to the USA Center for Disease Control and WHO.^35^

The true long term effect of disruption by the covid-19 pandemic will require decades to fully materialise, but vaccination coverage is now further disproportionately lower in resource limited countries. A worldwide vaccination report from 2021 estimated that among 18.2 million children who were not vaccinated, 12.8 million (70%) lived in middle income countries and five million (27%) in low income countries, and the risk of further sporadic and unpredictable outbreaks of vaccine preventable diseases in future years resulting from unknown gaps in population immunity seems certain.^22 36^

## New vaccine technologies

The covid-19 pandemic greatly accelerated vaccine science and in particular the advent of lipid nanoparticle mRNA vaccines alongside DNA based viral vectored platforms, collectively termed genetic vaccine technologies. Genetic vaccines work by supplying the host with the genetic material needed to synthesise the target protein in situ, conceptually making the host the final step in the vaccine manufacturing process of the protein needed for subsequent presentation to antigen presenting cells that trigger the downstream apparatus for lasting adaptive immunity. Lipid nanoparticle mRNA vaccines use pathogen derived mRNA encased within a fat bubble made up of only four lipids, without reliance on complex chemical conjugation steps or rate limited cell lines, that confer key advantages in vaccine production at speed and scale. Production lead times can be as short as 3-6 months, compared with 5-11 months for protein conjugate and 4-6 months for live attenuated vaccines.^2337^,^40^ So far, only two lipid nanoparticle mRNA vaccines have reached licensure; for the SARS-CoV-2 virus in 2021 and for respiratory syncytial virus in 2024. For viral vectors, such as human or simian adenovirus, vesicular stomatitis virus, and modified vaccinia virus Ankara, the number of licenced disease areas is also small and includes SARS-CoV-2, dengue, Ebola, smallpox, and mpox.

Genetic vaccines are well suited for viral targets because of the use of the host machinery for target pathogen protein synthesis with major histocompatibility complex co-expression similar to how the virus behaves in natural infection. In contrast, bacterial proteins are expressed by bacterial machinery and other factors, such as bacterial diversity, number of antigens needed for protection, and immunogenic polysaccharide targets, and are prohibitively complex for mRNA and viral-vector delivery systems. Studies on simultaneous vaccination with genetic vaccines, such as SARS-CoV-2 with seasonal influenza and respiratory syncytial virus vaccines, have provided favourable safety and immunogenicity data, and polyvalent mRNA vaccines are in development in clinical trials. Taken collectively, the advent of genetic vaccine technology will not change routine maternal and childhood immunisation programmes that require polyvalent vaccines and coverage of bacterial targets.

In learning lessons from the covid-19 pandemic, and more recently from the mpox and Marburg outbreaks in East Africa, genetic vaccine technologies are likely to be used first and early into the control of new viral pathogen disease outbreaks.^41^ Data from the Africa Centre for Disease Control showed that of the 1084.5 million covid-19 vaccine doses given across the continent up to September 2023, >80% were lipid nanoparticle mRNA or viral vectored genetic vaccine platforms.^42^,^44^ Key for future impact is how genetic vaccines require a special vaccine cold chain consideration. Unlike subunit and live attenuated vaccines, as used in the WHO Expanded Programme of Immunisation schedule, the long term stability of genetic vaccines requires storage at −20 °C or colder, and current vaccine cold chains designed around the demands before the covid-19 pandemic are not engineered for these conditions. When removed from −20 °C, genetic vaccine stability at fridge temperatures of 2-8°C is typically weeks to months, compared with 2-3 years for non-genetic vaccine counterparts (figure 2).^37 38^

Vaccine cold chain design to incorporate future genetic vaccine deployability not only requires ultra-cold capacity that currently does not exist in many vaccine cold chains in low and middle income countries, but also ultra-fast distribution systems to minimise the risk of vaccine damage and waste. Data on deployment of the covid-19 vaccine in Rwanda, one of only four African countries to achieve the WHO target of 70% population coverage by mid-2022, showed that 20 374 litres of vaccine freezer capacity out of a country vaccine cold chain total capacity (all fridges and freezers) of 245 481 litres (8.3% of total) underpinned this success (2022 audit data from Rwanda Biomedical Centre). These data show how vaccine cold chains from low and middle income countries can be adapted for genetic vaccine deployment given that many settings have limited freezer storage for vaccines in the WHO Expanded Programme of Immunisation schedule, such as oral poliovirus and measles-rubella vaccines.^45^

## New vaccines for outstanding global health priorities

The unmet burden of infectious diseases in low and middle income countries drives an expanding repertoire of vaccine countermeasures, all of which require capacity in already stretched vaccine cold chains.^39^ In the past 10 years, the control of major outstanding global health priorities has begun to be managed through newly licensed vaccines where none existed before. Vaccines include those for the prevention of typhoid (Vi tetanus toxoid, Bharat Biotech International), malaria (RTS,S/ASO1, GSK), respiratory syncytial virus (RSVPreF3, GSK and mRNA-1345, Moderna), as well as the many new vaccines used to accelerate the end of the covid-19 pandemic.

As the pipeline of experimental vaccine candidates makes scientific breakthroughs, the innovation to impact in low and middle income countries requires consideration of the vaccine cold chain systems in these countries. For example, the RTS,S/ASO1 vaccine for the prevention of malaria requires four doses (at ages five, six, seven, and 12-18 months), ideally before the malaria transmission season and in conjunction with the existing WHO Expanded Programme of Immunisation schedule. In African settings, transmission of respiratory syncytial virus occurs all year with little or no seasonal variation. Malaria and respiratory syncytial virus, however, are the first and second greatest single pathogens causing death between the first month and first year of life in all cause mortality estimates in low and middle income countries (11.8% and 6.7%, respectively), making them exceptionally relevant to African communities.^23 40^ The RTS,S/ASO1 malaria vaccine was approved by WHO in October 2021, became integrated into childhood immunisation programmes, and was prequalified in December 2023. This vaccine was systemically introduced in Cameroon in January 2024, and already an estimated 10.5 million doses have been deployed across 17 African nations.^40^ Maternal respiratory syncytial virus vaccines, as well as newly licensed long acting respiratory syncytial virus specific monoclonal antibodies for infants (nirsevimab, Sanofi) that also require vaccine cold chain functionalities, are yet to become available in African health economies.

## Vaccines to avoid the need for a vaccine cold chain

A longstanding objective of vaccine science has been to engineer thermotolerant vaccine products that do not require cold storage for preservation. In the interim, efforts have been made to develop and expand the number of products used in a variation of the vaccine cold chain, called the controlled temperature chain, for low and middle income countries.^46^,^48^ The aim has been to penetrate vaccine deployment into where little or no vaccine cold chain infrastructure can support, and typically as part of campaigns of supplementary immunisation activities.^49^ Currently, only a limited number of vaccine products are WHO prequalified for controlled temperature chain use and include meningococcal group A and ACWY conjugate vaccine, an oral cholera vaccine, some human papillomavirus vaccines, a combined tetanus toxoid-hepatitis B virus vaccine, and the typhoid conjugate vaccine, with expansion of the controlled temperature chain repertoire under development through the Vaccine Innovation Prioritization Strategy led by WHO, Unicef, Gavi, the Vaccine Alliance, the Bill and Melinda Gates Foundation, and PATH.^50 51^

Lyophilisation, or freeze drying, has improved heat stability and storage duration, and current research has focused on the use of cryoprotectants, such as deep eutectic solvents and carbohydrate polymers (of trehalose, sucrose, or gellan sugars) that can stabilise live viral vectors at 45 °C for six months, as well as encapsulation within heat resistant polymers and lipid nanoparticles, use of microneedle or microarray patches, vacuum or spray drying, and mRNA inserts of shorter nucleotide sequences that have shown promise in the search for improved thermal tolerance.^752^,^56^ The advent of AI may further unlock discoveries in vaccine antigen, adjuvant, and thermal stabilisation approaches.^57 58^

## Vaccine manufacturing

In the past 10 years, WHO has declared seven public health emergencies events of immediate concern, for H1N1 influenza virus (2009-10), Ebola virus disease (West Africa, 2013-16), poliomyelitis (2014-present), Zika virus (2015-16), Ebola virus disease (Democratic Republic of the Congo, 2018-20), SARS-CoV-2 virus (2020-23), and mpox (2024-25), with smaller outbreaks, such as Marburg virus (2024, Rwanda), and others that needed rapid vaccine deployment together with other vaccine provisions.^59^,^61^ Sub-Saharan Africa will likely have many more outbreaks of infectious diseases from known threats, as well as from unknown threats, such as disease X, a WHO term used to refer to a hypothetical new pathogen with epidemic or pandemic potential.

Even with efforts to expand regional and local production, low and middle income countries are reliant on international supplies that limit immunisation programme responsiveness. The top 10 vaccine manufacturers dominate 75% of the global vaccine market.^62^ The European Union leads with 44% of global exports (excluding trade within the European Union), followed by India (25%) and the US (15%). India, however, produces and exports 81% of the vaccines used in low income countries, based on data from 2017-19.^63^ Vaccine manufacturers commonly license their patents to producers in low income countries to sell to other low income countries, and the Serum Institute of India is currently the world's largest vaccine manufacturer, producing 1.5 billion doses each year (60% of the world supply), used in about 170 countries. Funding and other procurement mechanisms for provision of vaccines to low and middle income countries, such as those coordinated by Gavi, the Vaccine Alliance (2000-) and the Coalition of Epidemic Preparedness Innovations (2017-), map closely to global initiatives to ensure universal access to vaccines (WHO endorsed immunisation agenda 2030, IA2030), and to deal with immunity gaps resulting from covid-19 disruption (The Big Catchup, 2023-24), the growth of the WHO Expanded Programme of Immunisation to 17 vaccine preventable diseases, and many disease specific control and elimination initiatives. These initiatives have included bespoke targeting on polio (Global Polio Eradication Initiative, 1988), tetanus (Maternal and Neonatal Tetanus elimination, 1989), measles and rubella (Measles and Rubella Initiative, 2001), tuberculosis (End TB strategy, 2015), viral hepatitis (Global Health Sector strategy on viral hepatitis, 2016), malaria (Global Technical Strategy for Malaria, 2016), yellow fever (Eliminate Yellow Fever Epidemics strategy, 2017), human papillomavirus (Global Strategy to Accelerate the Elimination of Cervical Cancer, 2020), and others.^59 60^ As of 2025, Africa produces only about 1.1% of the vaccines used on the continent. The Africa Union has intensified sustainability and readiness for future outbreaks that has included the Partnerships for African Vaccine Manufacturing Framework for Action to produce 60% of the continent's vaccine requirements by 2040, as part of the Africa Union’s agenda 2063. Also, many African countries are preparing for mRNA production in partnership with the Coalition of Epidemic Preparedness Innovations, BioNTech, and others. Future African vaccine cold chains will need to accommodate this initiative.

## Climate change dynamics

The effects of climate change overlap with all aspects of vaccine cold chain vulnerabilities and resilience, and are most acute in already fragile low and middle income countries. Rising temperatures, excess precipitation (floods), and insufficient precipitation (droughts) across large geographical regions is a One Health emergency mediated by changes to disease vectors, environmental reservoirs of infection, and population migration and density.^61^ Disease vectors, such as *Anopheles*, *Aedes*, and *Culex* mosquitoes, *Ixodes* ticks, *Culicoides* flies, and others, transmit a heavy burden of infectious diseases in tropical and subtropical regions (WHO estimate of 17% of all infectious diseases).^64 65^ The epidemiology of vector borne diseases from protozoa (malaria, leishmaniasis, sleeping sickness, and filariasis), viruses (dengue, chikungunya, Zika, yellow fever, West Nile, Japanese encephalitis, Oropouche, and Rift Valley fever), helminths (schistosomiasis and hookworm), and others is likely to increase. Food and water borne vaccine preventable diseases (*Salmonella Typhi* and *Salmonella Paratyphi*, *Vibrio cholerae*, poliomyelitis, rotavirus, and others), collectively as diarrhoeal diseases, already account for 12% of all cause mortality in children aged 1-4 years (a rate comparable with malaria).^23^ Airborne infections are likely to change because of increasing population density, and erosion of seasonality, more common in high income countries, may diminish the effect of timing deployment of respiratory vaccines for annual infections of seasonal influenza, SARS-CoV-2 infection, and respiratory syncytial virus.^61^ African vaccine cold chain networks could easily find themselves in the wrong place and wrong time when most needed.

## Non-vaccine technologies

Investment in new technologies and the synergistic integration of these technologies, as well as the skilled workforce, will become an increasing factor in the future impact of vaccines in African low and middle income countries.^66^,^71^ Initiatives such as the Cold Chain Equipment Optimisation Platform and Gavi, the Vaccine Alliance ($250m, 2017-21), and other initiatives through Unicef and WHO, have co-invested with low and middle income countries to support access to high performance vaccine cold chain equipment.^71^ New WHO performance, quality, and safety approved fridge technologies can use AI optimised compressor efficiencies and energy demand prediction algorithms to consume 20-40% less energy than older units, and can use low global warming potential refrigerants, such as blends of R290 and R600a (propane and isobutane), instead of traditional hydrofluorocarbons.^72^ Small, fully solar powered WHO approved fridges can remove all of the risks and geographical restrictions associated with reliance on grid electricity infrastructure and long term energy costs.^1073^,^75^

For the vaccine cold chain network edges, unmanned aerial vehicles (or drones) and aerial logistics have been shown to be cost effective in the longer term, capable of supplying millions of vaccine doses (Nigeria, Ghana), reducing stockouts by 60% (Ghana), acting interchangeably across sector utility for veterinary disease interruption for Rift Valley fever protection (Rwanda), and can safely reduce the quantity of vaccine storage in vulnerable remote rural clinics by >90% in African low and middle income countries (Rwanda)^76^,^79^ (Rukundo et al, in submission, 2026). Technology can also help with embedding digital visibility, tracking, and blockchain accountability of vaccine stock through the whole of the vaccine cold chain network.^80 81^ Currently, smart labelling with GS1 powered QR codes and more advanced technologies, such as radio frequency identification, allow real time tracking and authentication of vaccine stock to the level of the primary packaging. A step further would be towards the unique labelling of individual vials for full visibility of open and closed vial losses and system inefficiencies as they disperse through the vaccine cold chain in real time.

Not only could this approach support the operational performance of public health institutions and non-governmental organisations, but the increasing problem of vaccine hesitancy in the public might also be helped. Vaccine hesitancy is a complex problem and often context specific and influenced by both social and scientific factors in Africa and elsewhere, representing a challenge to systematic and quantitative analyses.^8 82 83^ Concerns about counterfeit vaccines, or vaccines that have expired or been damaged by changes in temperature, however, can be mitigated by the use of vial specific digital product passports,^84^ as has been developed in other complex supply chains, so that individuals can verify the quality of product given to them or their child.

## Conclusions

The vaccine cold chain is a critical cooling and national infrastructure that underpins historical vaccine impact globally and sustains vaccine policy implementation and wide societal protections. System weaknesses, however, can lead to major losses in vaccine stock and failures to meet the needs of the population. This weakness is most severe in sub-Saharan African countries that carry the greatest burden of vaccine preventable disease and death. Current and future prospects include innovations that need routes to affect change in low and middle income countries as well as readiness for unprecedented and unpredictable threats to capacity and performance. To improve and sustain vaccine security, the next generation of vaccine cold chain systems for African communities needs to reach all communities in time for protection, to minimise waste, offer good value for money, and maintain the trust of the public. At the same time, African low and middle income countries must become ever more flexible to mitigate unexpected losses in capacity or peaks in demands driven by climate change, and use the latest innovations and advances in vaccine science, cooling technologies, transportation logistics, and digital tracking as priorities. The uncertainties of the future require strength in agility.

**Questions for future research**

- What and where are the best value for money investments for vaccine cold chain systems in low and middle income countries?
- How can standards for the cost effectiveness of the vaccine cold chain be measured and developed against different vaccine preventable diseases?
- How can existing operational performance data be leveraged to gain vaccine cold chain efficiencies?
- How can vaccine cold chain operations be made more visible and transparent to help with public confidence in health implementation agencies and taxpayer expenditure?
- Would innovations such as access to dose specific digital product passports help with vaccine hesitancy and decisions on whether to accept a vaccine?
- What are the future healthcare workforce skills needed for the advent of vaccine manufacturing in Africa and use of genetic vaccines?

**Patient involvement**

Patients and/or the public were not involved in the design, or conduct, or reporting, or dissemination plans of this research.
