# Correction: Vaccine cold chain and understanding what underpins vaccine security for vaccine preventable diseases

**DOI:** 10.1136/bmjmed-2025-001835corr1

**Published:** 2026-06-04

Rukundo G, Moore M, Semukunzi H, et al. Vaccine cold chain and understanding what underpins vaccine security for vaccine preventable diseases. *BMJ Med* 2026;5:e001835.

In this research article by Rukundo and colleagues (BMJMED 2026;5:e001835, doi:10.1136/bmjmed-2025-001835, published 9 April 2026), the final two labels at the bottom of figure 3 should have stated “9 months” and “15 months” rather than “9 weeks” and “15 weeks,” respectively. The article has been updated.

